# Parenting through grief: A cross-sectional study of recently bereaved adults with minor children

**DOI:** 10.1177/02692163211040982

**Published:** 2021-08-22

**Authors:** Eliza M Park, Allison M Deal, Justin M Yopp, Stephanie A Chien, Sean McCabe, Ariella Hirsch, Savannah M Bowers, Teresa Edwards, Donald L Rosenstein

**Affiliations:** 1 Department of Psychiatry, University of North Carolina at Chapel Hill, Chapel Hill, NC, USA; 2 Department of Medicine, University of North Carolina at Chapel Hill, Chapel Hill, NC, USA; 3 Lineberger Comprehensive Cancer Center, University of North Carolina at Chapel Hill, Chapel Hill, NC, USA; 4 Department of Biostatistics, University of North Carolina at Chapel Hill, Chapel Hill, NC, USA; 5 Department of Health Policy and Management, University of North Carolina at Chapel Hill, Chapel Hill, NC, USA; 6 H. W. Odum Institute for Research in Social Science, University of North Carolina at Chapel Hill, Chapel Hill, NC, USA

**Keywords:** Depression, bereavement, grief, parents, adaptation, psychological, widowhood

## Abstract

**Background:**

Grieving adults raising parentally-bereaved minor children experience persistently elevated symptoms of depression and grief. However, the factors associated with their mental health outcomes are not well understood.

**Aim:**

To investigate the psychosocial and demographic characteristics associated with grief distress and depressive symptom severity in bereaved adults with minor children.

**Design:**

Cross-sectional, web-based survey.

**Setting/participants:**

Eight hundred forty-five bereaved adults raising minor (age <18 years) children who had experienced the death of a co-parent. Primary outcomes were grief distress (Prolonged Grief Disorder-13), depressive symptoms (Patient-Reported Outcomes Measurement Information System-Depression), and widowed parenting self-efficacy (WPSES).

**Results:**

Mean grief scores were 33.5; mean depression scores were 58.3. Among the 690 individuals more than 6 months bereaved, 132 (19.3%) met criteria for prolonged grief disorder. In adjusted models, participants reporting higher grief scores were more recently bereaved, identified as mothers, non-Caucasian, had lower education and income, and had not anticipated their co-parent’s death. The statistical modeling results for depression scores were similar to grief scores except that depression was not associated with anticipation of co-parent death. Parents reporting lower WPSES scores had higher grief and depression scores. Retrospective assessments of more intense parenting worries at the time of co-parent death were also associated with higher grief and depression scores.

**Conclusions:**

For bereaved adults with minor children, unanticipated co-parent death was linked with higher grief distress but not symptoms of depression. Addressing parenting concerns may represent a common pathway for improving the mental health of parentally-bereaved families.

**What is already known about the topic?**

- Bereaved adults with minor children are at increased risk of developing depressive disorders.
- For parentally bereaved children, the surviving parent’s emotional health is both influential and predictive of the children’s psychological outcomes.

**What this paper adds:**

- Among bereaved adults raising minor children, women experience more depressive symptoms in bereavement than men.
- For bereaved adults raising minor children, the unanticipated death of a co-parent was associated with greater grief distress but not symptoms of depression.
- Lower parenting efficacy and higher parenting concerns in early bereavement were closely associated with poorer adjustment to bereavement in parents.

**Implications for practice, theory, or policy**

- Assessing parenting concerns in the early bereavement period could enhance the provision of family-centered end-of-life care for dying parents.
- Across the globe, the devastating toll of the COVID-19 pandemic creates a pressing need to support the mental health of newly bereaved adults with minor children.

## Background

The death of a parent creates profound disruption for families with minor (age <18 years) children. Surviving parents face the simultaneous challenges of coping with personal loss while parenting their grieving children,^1^ and the limited data available suggest that these bereaved adults suffer depressive symptoms that persist for extended periods of time.^2^ For parents and their children, bereavement is associated with increased psychiatric illness,^3–6^ suicidality,^7,8^ physical health problems,^6,9–11^ and increased mortality.^12,13^

Ongoing public health crises heighten the need to identify and intervene with parentally-bereaved families.^14,15^ In the United States, both suicide and the opioid epidemic affect young and middle aged adults during their prime parenting years.^15–17^ Globally, the COVID-19 pandemic caused the premature death of millions of adults in 2020 alone.^18^ The scale of the pandemic brings into bold relief the needs of bereaved parents and their children.

Several lines of research demonstrate the importance of identifying psychiatric illness in grieving parents and intervening for both their benefit and their children.^19^ Untreated depression in bereaved adults is associated with poorer physical health and psychiatric disorders in their children.^20–23^ Not only do such parents experience increased sadness and anxiety, they are less satisfied in their coping responses to parenting stressors, less accurate in their assessments of their children’s bereavement, and less aware of their children’s needs.^24^ It is the functioning of the surviving parent—rather than the circumstances of the parental death itself—that most influences the well-being and trajectory of children’s adjustment to loss.^25^ While there is compelling evidence documenting the needs of parentally-bereaved children, relatively little is known about the bereavement and psychosocial experiences of their grieving parents. Spousal status and the relationship to the deceased are frequently assessed in studies of bereavement, yet the parental status of the bereaved individual is commonly overlooked. Spousal or partner-loss is consistently found to be a risk factor for poor bereavement outcomes,^26^ yet the contribution of parenting concerns or challenges to grief outcomes is unknown. The few studies of bereaved adults with minor children primarily rely on qualitative data and are now decades old.^27–30^ How the bereavement experiences of these grieving adults may differ between mothers and fathers as well as the circumstances of the co-parent’s death have not been well described in the literature.^2^ Therefore, the objective of this study was to examine the psychosocial and demographic factors associated with grief distress and depressive symptoms in a cross-sectional sample of recently bereaved adults with minor children, using validated risk-assessment screening measures for prolonged grief disorder and depression. We focused on both grief and depressive outcomes because of the importance that parental depression symptoms have on children’s bereavement outcomes.^22,24,25,31^

## Method

### Study sample and design

We conducted a cross-sectional, web-based survey of the psychosocial, and grief experiences of adults raising parentally-bereaved children. The survey was available to all individuals who visited a public resource website for widowed parents (www.widowedparent.org). Participants did not receive any monetary (or equivalent) incentive for completing the survey.

Eligible individuals were adults at least 18 years of age who self-reported the death of a co-parent within the past 3 years and self-identified as the primary caregiver of a parentally-bereaved minor child at the time of parental death. We defined a co-parent as an adult who was also a parent to the child(ren). Individuals did not have to be partnered or legally married to the deceased parent at the time of death. Data were collected from December 2017 to July 2019 using Qualtrics software (Qualtrics, LLC, Provo, UT, USA).

### Ethics

Participants provided electronic informed consent prior to starting the survey. All study procedures were approved by the University of North Carolina at Chapel Hill Institutional Review Board (#17-2546, 11/17/17).

### Survey overview

The 107 survey items queried grief distress, depressive symptoms, parenting self-efficacy and satisfaction, assessment of child traumatic stress, dependency on the bereaved, and psychological attachment to the bereaved. The survey was informed by previously published surveys of widowed parents and a review of the literature on bereaved adults and parentally-bereaved children.^2^ The survey was pretested with a sample of bereaved men and women with minor children and modified iteratively to enhance clarity, face validity, and content validity. The survey (available in the Supplemental Appendix) took a median of 15 minutes to complete and included adaptive questioning to decrease response burden. The survey also contained built-in checks to confirm participant eligibility.

### Survey domains

#### Grief distress and depression

Grief distress was measured by the Prolonged Grief Disorder-13 (PG-13). The PG-13 is a grief assessment tool used to identify prolonged grief disorder for individuals bereaved for at least 6 months or as a measure of grief distress when scored continuously. The PG-13 corresponds to the diagnostic criteria for prolonged grief disorder in the upcoming International Classification of Diseases 11th Revision, which includes substantial and persistent grief-related distress causing significant impairment in functioning.^32^ Total score range for the PG-13 is 11–55 with higher scores indicating higher distress. Prior studies have reported mean PG-13 scores of 27 and 48 as reflecting moderate and high risk for prolonged grief disorder, respectively.^33,34^

Depressive symptoms were assessed by the Patient Reported Outcomes Measurement Information System Depression 4-item short form (PROMIS-Depression).^35^ Responses to PROMIS measures are standardized to a mean of 50 (standard deviation, SD = 10) and normed to the United States population. We limited PROMIS-Depression analyses to those with complete measures.

#### Parenting self-assessments

Parenting self-efficacy was measured by the Widowed Parent Self-Efficacy Scale (WPSES), a 9-item Likert scale assessing widowed parents for parenting burden, parental expectations, and discipline of their children.^36^ The WPSES ranges from 0 to 6, with higher scores indicating higher self-efficacy.

Parenting satisfaction was assessed by the 3-item Kansas Parental Satisfaction Scale (KPSS). The KPSS is scored from 3 to 21, with higher scores indicating higher satisfaction.^37^

Investigator-designed questions addressed current and prior communication challenges with their children related to the co-parent’s death and the participant’s concerns immediately following the death of their co-parent (e.g. “In the weeks following [your co-parent’s] death, how much were you worried about the following topics?”). These concerns were presented as statements with a four-point ordinal response scale (0 = “Not at all” to 3 = “A lot”).

#### Relationship with the deceased

Participants’ emotional and physical dependency on the deceased were measured by the 6-item Likert scale, the Bereavement Dependency Scale (score range: 6–30, higher scores indicate more dependency).^38^ Participants’ psychological attachment to the deceased was measured by the 11-item Likert scale, the Continuing Bonds Scale (score range: 11–55, higher scores reflect higher continued bond with the deceased).^39^

#### Parental assessment of child stress

The Child Stress Disorders Checklist–Short Form (CSDS) is 4-item, parent-reported measure of traumatic stress in children.^40^ The score range is 0–8, with higher scores indicating greater risk for stress disorders. For families with more than one minor child, the participant was asked to select the child for whom they had the greatest concern.

### Participant characteristics

The survey included questions assessing demographic characteristics of the participant, their child(ren), and the deceased parent.

### Statistical analysis

Descriptive statistics were used to characterize the sample of bereaved adults. Scores for outcome measures were calculated according to individual scale instructions. Linear regression modeling assessed relationships between all demographic and family variables and the primary outcome measures of interest: the PG-13 (grief distress) and PROMIS-Depression (depressive symptoms). Backward selection (using a cut-off of *p* = 0.02) was used to identify the set of patient clinical and demographic characteristics important to control for in adjusted modeling. The final set of variables are listed in the Supplemental Table. The set was slightly different for the two outcome measures, thus, the sample sizes in the tables vary somewhat across results. All analyses were performed using SAS version 9.4 (SAS Institute, Inc., Cary, NC). All *p* values were derived from two-sided statistical tests, and no adjustments for multiplicity are included.

## Results

### Participant characteristics

A total of 1067 bereaved adults started the survey. Of these individuals, 845 met all eligibility criteria (Supplemental Figure 1). Table 1 describes characteristics of the participants, their children, and the deceased. Mean time between co-parent death and survey completion was 16.6 months (SD 10.2). Most participants were romantically partnered with the deceased parent at time of death (*N* = 742, 93.7%). The participant sample was nearly evenly split between individuals who reported at least some awareness or anticipation of their co-parent’s death (*N* = 400, 47.3%) and individuals who reported they “had no warning” (*N* = 445, 52.7%).

**Table 1.** Participant, child, and decedent characteristics.

Characteristic	Total (*n* = 845) *N* (%)
Decedent	
Cause of death	
Cancer	312 (36.9%)
Heart disease	116 (13.7%)
Other chronic illness	31 (3.7%)
Unintentional injury	157 (18.6%)
Suicide	111 (13.1%)
Other sudden illness	79 (9.3%)
Other cause^a^	36 (4.3%)
Death was anticipated by participant^b^	
Yes	400 (47.3%)
No	445 (52.7%)
Participant	
Age in years, mean (SD)	41.9 (8.3%)
Relationship with decedent in years, mean (SD)	15.7 (7.5%)
Period of bereavement	
Less than 6 months	153 (18.2%)
6–11 months	169 (20.0%)
12–17 months	146 (17.3%)
18–23 months	135 (16.0%)
24–36 months	240 (28.5%)
Gender	
Mother (women)	672 (79.5%)
Father (men)	173 (20.5%)
Race	
Caucasian	654 (84.3%)
Non-Caucasian	122 (15.7%)
Education	
Less than college degree	207 (26.5%)
College degree or higher	574 (73.5%)
Employed outside the home^c^	
Yes	536 (74.4%)
No	184 (25.6%)
Annual household income at time of survey	
Less than $50,000	326 (43.5%)
Greater than or equal to $50,000	423 (56.5%)
Current sole caregiver	
Yes	730 (92.3%)
No	61 (7.7%)
Nationality^d^	
United States	633 (89.7%)
Other country	73 (10.3%)
Importance of religious faith	
Not important at all	274 (34.7%)
Somewhat important	239 (30.3%)
Very important	276 (35.0%)
Child	
Number of children at home aged <18 years at time of co-parent death, mean (SD)	1.9 (1.0%)
Age of youngest child in years, mean (SD)	6.7 (4.8%)
Age of all minor children in years, mean (SD)	8.2 (4.7%)

Values are numbers (percentages) unless stated otherwise.

a Cause of death was re-categorized if participant description for “other” matched an existing response category. Data missing for one participant.

b Anticipated death defined as realizing that co-parent might die, anywhere from “less than 2 weeks” to “more than 2 years.” Unanticipated deaths defined as having “no warning” prior to the co-parent’s death that the co-parent might die.

c Defined as part- or full-time employment outside of the home.

d *N* = 706, 20 countries included in sample, those with >10 respondents included Australia (*N* = 15), Canada (*N* = 27), and the United Kingdom (*N* = 14).

### Grief distress and depressive symptoms

The mean PG-13 score for the sample was 33.5 (SD 9.4). Among respondents at least 6 months bereaved (*N* = 690, 81.8%), 132 (19.3%) met scoring criteria for prolonged grief disorder. The standardized mean PROMIS-Depression scores was 58.3 (SD 8.6) which corresponds to mild depression.^41^ Table 2 shows PG-13 and PROMIS-Depression scores for the sample as well as scores for all other measures.

**Table 2.** Mean and standard deviations for parent and child psychosocial adjustment measures.

Domain (scale)	*N*	Mean (SD)	Observed Range
Grief distress (PG-13)^a^	845	33.5 (9.4)	11.0–55.0
Depression symptoms (PROMIS-D)	838	58.3 (8.6)	41.0–79.4
Parenting self-efficacy (WPSES)	797	3.5 (0.9)	1.1–6.0
Traumatic stress in child (CSDS)^b^	788	2.0 (1.9)	0.0–8.0
Parenting satisfaction (KPSS)	772	14.5 (3.6)	3.0–21.0
Dependency on bereaved (BDS)	366	17.0 (4.5)	6.0–30.0
Attachment to bereaved (CBS)	359	43.4 (9.4)	11.0–55.0

PG-13: Prolonged Grief-13 Tool; PROMIS Depression: Patient-Reported Outcomes Measurement Information System—Depression; CSDS: Child Stress Disorders Checklist; WPSES: Widowed Parenting Self-Efficacy Scale; KPSS: Kansas Parental Satisfaction Scale; BDS: Bereavement Dependency Scale; CBS: Continuing Bonds Scale.

a Imputed missing items using respondents’ mean scores if they completed at least nine items (*N* = 16).^34^

b For families with more than one minor child, the participant was asked to select the child for whom they had the greatest concern.

### Demographic characteristics associated with grief distress and depressive symptoms

Women were more likely than men to report symptoms of grief distress (mean 34.2 vs 30.6, *p* < 0.0001) and depression (59.0 vs 55.6, *p* < 0.0001). In unadjusted analyses, individuals who experienced the unanticipated death of their co-parent also experienced heightened grief distress (35.5 vs 31.2, *p* < 0.0001) and depression (59.6 vs 56.7, *p* < 0.0001) scores. Grief distress was incrementally associated with duration of bereavement. Individuals less than 6 months bereaved reported the highest mean PG-13 scores (36.0). Mean PG-13 scores were 35.0 for individuals 6–11 months bereaved, 33.5 for individuals 12–17 months bereaved, and 32.7 for individuals 18–23 months bereaved. Individuals who were 24–36 months bereaved reported the lowest PG-13 scores (31.2) overall. Table 3 shows the bivariate associations for PG-13 and PROMIS-Depression scores with participant and family demographic characteristics.

**Table 3.** Unadjusted associations between participant and child demographic characteristics with grief distress and depression symptom scores.

Characteristic	Grief Distress (PG-13) (*N* = 845)		Depression symptoms (PROMIS-D) (*N* = 838)	
	β (95% CI)^a^	*p*-Value	β (95% CI)	*p*-Value
Anticipated death^b^	−4.36 (−5.59 to −3.12)^a^	<0.001	−2.83 (−3.99 to −1.68)^a^	<0.001
Years since death	−2.20 (−2.93 to −1.47))^a^	<0.001	−1.00 (−1.68 to −0.32)^a^	0.004
Parent age	−0.21 (−0.29 to −0.14)	<0.001	−0.20 (−0.27 to −0.13)^a^	<0.001
Father^b^	−3.58 (−5.13 to −2.02^a^	<0.001	−3.41 (−4.84 to −1.99)^a^	<0.001
Caucasian	−4.18 (−5.98 to −2.38)^a^	<0.001	−2.74 (−4.43 to −1.05)^a^	0.002
College degree or higher	−3.66 (−5.14 to −2.18)^a^	<0.001	−3.72 (−5.08 to −2.36)^a^	<0.001
Annual household income ⩾$50,000	−2.99 (−4.34 to −1.64)^a^	<0.001	−3.40 (−4.63 to −2.17)^a^	<0.001
Employed outside the home	−2.22 (−3.79 to −0.64)^a^	0.006	−1.11 (−2.58 to 0.37)	0.140
Length of relationship with co-parent	−0.12 (−0.21 to −0.03)	0.007	−0.15 (−0.23 to −0.07)	<0.001
Mean age of youngest child in years	−0.17 (−0.31 to −0.03)	0.016	−0.18 (−0.31 to −0.05)	0.008
Mean age of minor children in years	−0.15 (−0.29 to −0.01)	0.040	−0.15 (−0.28 to −0.01)	0.030
Sole caregiver	0.45 (−2.00 to 2.91)	0.72	−1.01 (−3.28 to 1.25)	0.380
Importance of religious faith^c^				
Somewhat important	0.05 (−1.58 to 1.68)	0.95	0.03 (−1.20 to 1.81)	0.690
Very important	−1.42 (−2.99 to 0.16)	0.08	−1.42 (−2.86 to 0.03)	0.054

PG-13: Prolonged Grief-13 Tool; PROMIS-D: Patient-Reported Outcomes Measurement Information System—Depression.

a Indicates variable was retained in the final model.

b Wilcoxon Rank Sum test used for non-parametric data.

c Referent: Not important.

Supplemental Table 1 lists the set of patient demographic and clinical characteristics that best explained the variation in scores. The following variables were associated with higher PG-13 scores, reflecting higher grief distress: unanticipated death, less time since death, female gender, non-Caucasian race, lower education, and lower household income. Modeling for PROMIS-Depression scores revealed overall similar results, except for unanticipated co-parent death which was strongly related to grief distress scores but not depression. In the final multivariable model, female gender; non-Caucasian race; lower education; lower household income; and less time since death were linked with higher PROMIS-Depression scores.

### Grief distress, depression symptoms, and parenting

After evaluating the relationships between demographic characteristics and both PG-13 and PROMIS-Depression scores, we examined the relationships of these outcomes with other psychosocial characteristics: widowed parenting self-efficacy, parenting satisfaction, current symptoms of traumatic stress in their child, dependency on the bereaved, and psychological attachment to the bereaved. Each of these structured measures were strongly associated with both PG-13 and PROMIS-Depression scores in unadjusted models and remained significant after adjustment for demographic characteristics. The differences in the unadjusted and adjusted models were minimal, therefore only adjusted model results are reported and shown in Table 4.

**Table 4.** Adjusted associations between measures of participant and child psychosocial adjustment with grief distress and depression symptom scores.

Domain (scale)	Grief distress (PG-13)^a^		Depression symptoms (PROMIS-D)^b^	
	*N*	β (95% CI)	N	β (95% CI)
Parenting self-efficacy (WPSES)	684	−4.23 (−4.87 to −3.58)**	736	−4.69 (−5.26 to −4.13)**
Traumatic stress in child (CSDS)^c^	675	1.07 (0.73 to 1.41)**	727	0.98 (0.67 to 1.29)**
Parenting satisfaction (KPSS)	680	−0.79 (−0.97 to −0.62)**	732	−0.95 (−1.11 to −0.8)**
Dependency on bereaved (BDS)	323	0.68 (0.47 to 0.89)**	341	0.67 (0.48 to 0.86)**
Attachment to bereaved (CBS)	314	0.28 (0.17 to 0.38)**	346	0.17 (0.07 to 0.26)*

PG-13: Prolonged Grief-13 Tool; PROMIS-D: Patient-Reported Outcomes Measurement Information System—Depression; CSDS: Child Stress Disorders Checklist; WPSES: Widowed Parenting Self-Efficacy Scale; KPSS: Kansas Parental Satisfaction Scale; BDS: Bereavement Dependency Scale; CBS: Continuing Bonds Scale.

a Adjusted for participant gender, race, education, income, employment status, time since death, and whether co-parent death was anticipated by participant.

b Adjusted for participant gender, participant age, race, education, income, time since death, and whether co-parent death was anticipated by participant.

c For families with more than one minor child, the participant was asked to select the child for whom they had the greatest concern.

* *p* < 0.001. **p < 0.0001.

### Early bereavement parenting concerns

**Figure 1.**
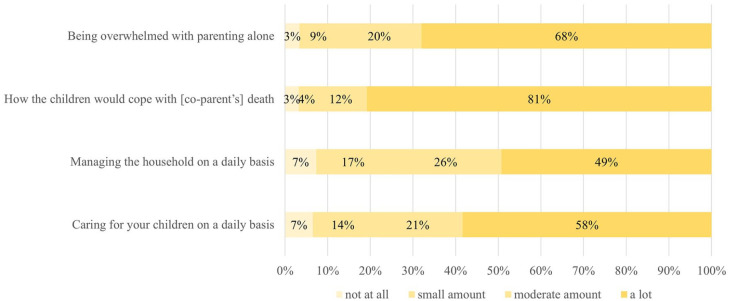
In the weeks following (co-parent’s death), how much were you worried about the following topics?

Parenting-related worries were prominent in the early bereavement period; 81% (*N* = 679) of the sample reported experiencing “a lot” of worry about how their children would cope following parental death. Most participants reported they had at least moderate worry about their ability to care for their children on a daily basis (*N* = 668, 79.3%) and being overwhelmed with parenting alone (*N* = 741, 87.8%). Survey results for the parenting concerns question items are shown in Figure 1.

There were no significant differences in PG-13 or PROMIS-Depression scores between the 27 individuals who reported “no worry” about how their children would cope with parental death and the 679 individuals who reported “a lot” of worry. In contrast, individuals who reported they had experienced “a lot” of worry for other parenting topics had higher PG-13 and PROMIS-Depression scores at the time of completing the survey. Participants who reported they had experienced “a lot” of worry about parenting alone had PG-13 scores 5.5 points higher than those who reported “no worry at all.” Multivariable regression analyses revealed that no individual parenting concern was predominantly associated with grief distress or depression (Table 5).

**Table 5.** Adjusted associations between parenting distress in early bereavement with grief distress and depression symptom scores.

Characteristic^a^	Grief distress (PG-13^b^) (*N* = 845)		Depression symptoms (PROMIS-D^c^) (*N* = 838)	
	β (95% CI)	*p*-Value	β (95% CI)	*p*-Value
Daily childcare				
Small amount	−0.28 (−3.35 to 2.78)	0.855	−0.63 (−3.39 to 2.13)	0.655
Moderate amount	1.20 (−1.68 to 4.07)	0.415	1.21 (−1.39 to 3.82)	0.361
A lot	4.94 (2.29 to 7.59)	<0.001	4.07 (1.67 to 6.46)	0.001
Managing household				
Small amount	1.00 (−1.82 to 3.83)	0.488	−0.75 (−3.32 to 1.83)	0.570
Moderate amount	−0.02 (−2.68 to 2.65)	0.991	−0.17 (−2.62 to 2.27)	0.890
A lot	4.42 (1.91 to 6.94)	0.001	2.97 (0.67 to 5.28)	0.011
Children coping				
Small amount	0.95 (−4.27 to 6.17)	0.720	2.42 (−2.20 to 7.05)	0.305
Moderate amount	−1.94 (−6.20 to 2.32)	0.373	−0.41 (−4.22 to 3.39)	0.832
A lot	1.86 (−1.97 to 5.69)	0.341	2.33 (−1.10 to 5.75)	0.184
Parenting alone				
Small amount	0.34 (−3.90 to 4.57)	0.877	0.06 (−3.74 to 3.85)	0.977
Moderate amount	0.85 (−3.07 to 4.76)	0.672	0.63 (−2.84 to 4.10)	0.724
A lot	5.49 (1.77 to 9.21)	0.004	4.62 (1.34 to 7.90)	0.006

PG-13: Prolonged Grief-13 Tool; PROMIS-D: Patient-Reported Outcomes Measurement Information System—Depression.

a For all categories, “not at all” was used as referent.

b Adjusted for participant gender, race, education, income, employment status, time since death, and whether co-parent death was anticipated by participant.

c Adjusted for participant gender, participant age, race, education, income, time since death, and whether co-parent death was anticipated by participant.

## Discussion

### Main findings

To our knowledge, this is the largest study to date to examine the mental health of grieving adults raising parentally-bereaved minor children. Results from this cross-sectional survey add to our understanding of the potential risk factors and clinical needs of parentally-bereaved families in several ways. First, this study augments prior research on the vulnerability of bereaved adults, particularly those who are widowed, to prolonged grief disorder and expands current research on characteristics of bereaved adults at risk for poor mental health outcomes.^26,42^ Although loss and grief are universal experiences, longitudinal assessments of bereaved adults suggest that only 10% experience prolonged grief disorder or other persistent psychiatric problems.^43^ Existing research suggests that individuals most likely to report needing professional and/or community-based grief support are young and middle aged adults.^33,44^ Unfortunately, these studies have not specifically evaluated parental status. The added challenges of parenting a grieving child may contribute to the vulnerability of these individuals who seek mental health support. Our findings suggest the need to further address how the parental role interrelates with age and risk of poorer grief adjustment.

Stroebe and Schut’s Dual Process Model of Coping with Bereavement serves to contextualize these results.^45^ The Dual Process Model posits that bereaved individuals face two post-loss psychological challenges: loss-oriented stressors (“grief work”) and restoration-oriented stressors (coping with future-oriented tasks). Healthy adaptation is contingent on oscillating attention and effort between these stressors. This may be especially challenging for adults grieving a premature death while meeting increased parenting demands at home. These individuals must also consider how their bereavement needs may differ from their children’s. For example, whereas children may benefit from a sustained attachment to their deceased parent, this dynamic may not be adaptive for bereaved adults for whom continuing bonds are associated with prolonged symptoms of grief.^25,39^ In this study, participants’ report of a stronger continuing bond with the deceased was linked to more depressive symptoms and grief. How parents balance their own grief work with that of their children remains unclear but may limit their ability to simultaneously meet both needs.^39^

The results of this study suggest several clinical applications. The WPSES, which assesses perceived parenting efficacy of widowed parents, demonstrated strong associations with both grief and depression. Lower parenting self-efficacy was also linked to female gender and unexpected loss—factors independently associated with higher risk of grief distress. Parents who believe that they are inadequately meeting their grieving children’s needs could also be more vulnerable to psychiatric disorders (or vice versa), which in turn could interfere with child-centered parenting practices. While the cross-sectional nature of this study does not allow us to draw conclusions about the causality of these likely bidirectional relationships, they do suggest a compelling role for the early assessment of parenting self-efficacy in bereavement or even prior to parental death.^46^

While grief is an intensely individualized experience, a better understanding of the characteristics associated with poorer adjustment will clarify the societal and familial costs of prolonged grief and associated mood disorders. Results from this study also suggest that sociodemographic characteristics of these individuals may place them at increased risk for grief complicated by co-occurring depression. Prolonged grief disorder and bereavement-related depressive disorders are diagnostically distinct entities that benefit from different treatment approaches,^47^ yet their complex relationship can challenge clinical management. Addressing the parental role may represent a way to help individuals with both disorders. Assessing for parenting-related distress in the early bereavement period and offering services for newly complex parenting needs is a potential way of offering targeted mental health support for at-risk individuals.

### Strengths and limitations

Our study results are based on cross-sectional data; thus, we cannot assign a causal role to the factors associated with psychosocial adjustment. The individuals who completed the survey were not drawn from a population-based sample. Much of the bereavement literature relies on self-selected samples or individuals attending bereavement support programs, which each introduce their own potential sampling biases.^43,48^ We know little about the individuals who accessed the website and chose not to participate. At the same time, this study did not seek to establish prevalence estimates of depression or prolonged grief disorder, but rather to better understand the parenting-related factors that related to the experience of post-loss depressive and grief symptoms.

Beyond these limitations, the strengths of this study are noteworthy. The survey included several detailed questions specific to parentally-bereaved families and the relatively large sample size allowed for the inclusion of potential covariates and greater precision of estimates for multivariable analyses. Demographic characteristics such as female gender and lower household income, and bereavement characteristics such as unanticipated death were independently and collectively associated with worse outcomes. The characteristics described above are readily identifiable at the time of death and lend support for proactive intervention for these families. These results can inform end-of-life care, suggest potential windows for intervention for at-risk families, and allow clinicians and researchers to better understand the impact of parental death on surviving family members.

## Conclusion and future directions

As a hypothesis-generating study, our findings suggest a need for further exploration. Future analyses from this study will assess parenting-specific end-of-life characteristics. Studies utilizing prospective assessments may help build a framework for how and when parental status impacts the bereavement trajectory and long-term adjustment of surviving family members. Greater understanding of the additional challenges and experiences that individuals in same sex partnerships, racial and ethnic minorities, migrant and refugee populations, and non-parental caregivers are needed. Unsurprisingly, identifying and recruiting individuals from these populations is challenging given the lack of sustained contact between surviving family members and the healthcare providers for the deceased.^49^

Families affected by the premature death of a parent face serious challenges in early bereavement. While a growing evidence-base supports the role for intervening with parentally-bereaved children, the needs of their grieving parents are overlooked. Worldwide, millions of adults newly face widowed parenthood and the COVID-19 pandemic will surely lead to more.^14^ Alleviating the long-term mental health consequences of unanticipated bereavement may require careful attention to the parental role.

## Supplemental Material

sj-docx-3-pmj-10.1177_02692163211040982 – Supplemental material for Parenting through grief: A cross-sectional study of recently bereaved adults with minor children Click here for additional data file. Supplemental material, sj-docx-3-pmj-10.1177_02692163211040982 for Parenting through grief: A cross-sectional study of recently bereaved adults with minor children by Eliza M Park, Allison M Deal, Justin M Yopp, Stephanie A Chien, Sean McCabe, Ariella Hirsch, Savannah M Bowers, Teresa Edwards and Donald L Rosenstein in Palliative Medicine

sj-pdf-1-pmj-10.1177_02692163211040982 – Supplemental material for Parenting through grief: A cross-sectional study of recently bereaved adults with minor children Click here for additional data file. Supplemental material, sj-pdf-1-pmj-10.1177_02692163211040982 for Parenting through grief: A cross-sectional study of recently bereaved adults with minor children by Eliza M Park, Allison M Deal, Justin M Yopp, Stephanie A Chien, Sean McCabe, Ariella Hirsch, Savannah M Bowers, Teresa Edwards and Donald L Rosenstein in Palliative Medicine

sj-tif-2-pmj-10.1177_02692163211040982 – Supplemental material for Parenting through grief: A cross-sectional study of recently bereaved adults with minor children Click here for additional data file. Supplemental material, sj-tif-2-pmj-10.1177_02692163211040982 for Parenting through grief: A cross-sectional study of recently bereaved adults with minor children by Eliza M Park, Allison M Deal, Justin M Yopp, Stephanie A Chien, Sean McCabe, Ariella Hirsch, Savannah M Bowers, Teresa Edwards and Donald L Rosenstein in Palliative Medicine
