# Supplementary material for: Parenting through grief: A cross-sectional study of recently bereaved adults with minor children
Source: Palliat Med. 2021 Aug 22;35(10):1923–32. doi: 10.1177/02692163211040982 (PMC8637383; doi:10.1177/02692163211040982)
Supplement: sj-docx-3-pmj-10.1177_02692163211040982 – Supplemental material for Parenting through grief: A cross-sectional study of recently bereaved adults with minor children [file sj-docx-3-pmj-10.1177_02692163211040982.docx]

| **Supplemental Table.** Multivariable regression model for grief distress and depression symptom scores | | | | |
| --- | --- | --- | --- | --- |
|  | **Grief Distress**  **(PG-13)**  (N = 845) | | **Depression symptoms (PROMIS-D )**  (N = 838) | |
| **Characteristic** | **β (95% CI)** | **P value** | **β (95% CI)** | **P value** |
| Anticipated death | -3.44 (-4.85 to -2.02) | <0.0001 | -1.19 (-2.48 to 0.10) | 0.070 |
| Years since death | -2.55 (-3.35 to -1.76) | <0.0001 | -1.25 (-1.98 to -0.52) | 0.001 |
| Parent age | -- |  | -0.06 (-0.14 to 0.02) | 0.152 |
| Father | -2.37 (-4.12 to -0.62) | 0.0079 | -2.69 (-4.33 to -1.06) | 0.001 |
| Caucasian | -2.91 (-4.74 to -1.08) | 0.0018 | -1.74 ( -3.39 to -0.08) | 0.040 |
| College degree or higher | -1.62 (-3.26 to 0.01) | 0.0515 | -2.28 (-3.72 to -0.83) | 0.002 |
| Annual household income ≥ $50,000 | -1.57 (-3.06 to -0.08) | 0.038 | -1.95 (-3.29 to -0.62) | 0.004 |
| Employed outside the home | -1.24 (-2.81 to 0.34) | 0.123 | -- |  |
| PG-13=Prolonged Grief-13 Tool; PROMIS-D=Patient-Reported Outcomes Measurement Information System - Depression | | | | |
