# Supplementary material for: Parenting through grief: A cross-sectional study of recently bereaved adults with minor children
Source: Palliat Med. 2021 Aug 22;35(10):1923–32. doi: 10.1177/02692163211040982 (PMC8637383; doi:10.1177/02692163211040982)
Supplement: sj-pdf-1-pmj-10.1177_02692163211040982 – Supplemental material for Parenting through grief: A cross-sectional study of recently bereaved adults with minor children [file sj-pdf-1-pmj-10.1177_02692163211040982.pdf]

SUPPLEMENTAL APPENDIX

Widowed Parent Survey

1. Are you a mother or father?	○ Mother<br>○ Father
2. Was your child(ren)'s deceased parent a mother or a father?	○ Mother<br>○ Father
3. How would you like us to refer to your co-parent for the remainder of the survey? Please provide a first name, initials, or nickname.	______________________
4. What was the cause of [*Name*]'s death?	○ Cancer<br>○ Heart disease (for example, a heart attack)<br>○ Other chronic illness (for example, diabetes)<br>○ Other sudden illness (for example, pneumonia)<br>○ Unintentional injury (for example, motor vehicle accidents or accidental overdose)<br>○ Suicide<br>○ Other (please describe)
5. How long before [*Name*] died did you realize that [he/she] might die?	○ I had no warning (for example, a sudden death due to car accident or heart attack)<br>○ Less than two weeks<br>○ Two weeks to one month<br>○ Between one to six months<br>○ More than six months<br>○ More than one year<br>○ More than two years
The next few questions are about [Name]'s death.	
6. When did [*Name*] die?	Month: [dropdown list of months]<br>Year: [allowed range 2015 – 2030]
7. How old was [*Name*] when [he/she] died?	________________ years
8. Where did [*Name*] die?<br>**[ONLY SHOW IF Q5 = Response 2-7]**	○ In a hospital<br>○ At home<br>○ In a standalone or separate hospice facility<br>○ Elsewhere, please describe
9. Did [*Name*] die in the location [he/she] had chosen?<br>**[ONLY SHOW IF Q8 ASKED]**	○ Yes<br>○ No<br>○ Unsure<br>○ She/he had not expressed a location preference

10. Did [*Name*] receive services from hospice? **[ONLY SHOW IF Q8= Response 1, 2, 4]**

- ○ Yes
- ○ No
- ○ Unsure

11. How long did [*Name*] receive hospice services (inpatient and/or at home)?

- ○ Less than 3 days
- ○ Between 3-7 days
- ○ Between 1 week and 1 month
- ○ More than one month
- ○ More than three months

Thinking about [Name], how true would you say each of the following statements is? Please select one response on each row. **[ONLY SHOW IF Q5 = Response 2-7]**

	Very True	Mostly True	A little true	Not true at all	Unsure
12. [*Name*] was at peace with dying	○	○	○	○	○
13. [*Name*] was afraid of dying	○	○	○	○	○
14. [*Name*] was worried that parenting alone would be overwhelming for you	○	○	○	○	○
15. You and [*Name*] had said 'goodbye' to each other	○	○	○	○	○
16. [*Name*] and the child(ren) had said 'goodbye' to each other	○	○	○	○	○
17. [*Name*] had [his/her] funeral arrangements in order	○	○	○	○	○

Below is a list of topics that some parents discuss when they know that one parent's life is coming to an end. First we are going to ask you ***how much*** *you and [Name] discussed the following topics before [he/she] died.*

In the next section, we are going to ask you ***how well you understand*** *[Name's] wishes on the* ***same topics.*** *We recognize that sometimes people do not discuss certain issues with their co-parent because they feel like they already know their co-parent's opinion.* **[ONLY SHOW IF Q5 = Response 2-7]**

How much *did you and [Name] discuss the following things before [he/she] died?*

	Not at all	A small amount	A moderate amount	A lot
18. Whether/how to expose the child(ren) to the dying process	○	○	○	○
19. Raising the child(ren) in ways that reflect [*Name*]'s wishes/values/hopes	○	○	○	○
20. Whether/how to talk with the child(ren) about [*Name*]'s impending death	○	○	○	○
21. Whether/how to talk with the child(ren) about [*Name*]'s death after [he/she] died	○	○	○	○
22. Whether you might date or remarry	○	○	○	○
23. How you would manage the household on a day-to-day basis	○	○	○	○

24. Family finances (for example, bank accounts, health insurance)	○	○	○	○
25. Whether/how to maintain family traditions	○	○	○	○
26. Whether/how you and/or the child(ren) would maintain contact with [*Name*]'s parents, siblings, or other family after [his/her] death	○	○	○	○

***How well do you feel you understand** [Name]'s wishes on the **same topics**?*

	Not at all	A small amount	A moderate amount	A lot
27. Whether/how to expose the child(ren) to the dying process	○	○	○	○
28. Raising the child(ren) in ways that reflect [*Name*]'s wishes/values/hopes	○	○	○	○
29. Whether/how to talk with the child(ren) about [*Name*]'s impending death	○	○	○	○
30. Whether/how to talk with the child(ren) about [*Name*]'s death after he/she died	○	○	○	○
31. Whether you might date or remarry	○	○	○	○
32. How you would manage the household on a day-to-day basis	○	○	○	○
33. Family finances (for example, bank accounts, health insurance)	○	○	○	○
34. Whether/how to maintain family traditions	○	○	○	○
35. Whether/how you and/or the child(ren) would maintain contact with [*Name*]'s parents, siblings, or other family after [his/her] death	○	○	○	○

36. Are there topics that you and [*Name*] did not discuss prior to [his/her] death that you wish you had?	○ Yes<br>○ No **[SKIP TO Q38]**
37. What would you say are the most important topics that you and [*Name*] did not discuss prior to [his/her] death that you wish you had?	

In the weeks following [Name]'s death, how much were you worried about the following topics? Please select one response on each row.

	Not at all	A small amount	A moderate amount	A lot
38. Financial concerns	○	○	○	○
39. Caring for your children on a daily basis	○	○	○	○
40. Managing the household on a day-to-day basis	○	○	○	○
41. Your own health	○	○	○	○
42. How the children would cope with [*Name*]'s death	○	○	○	○
43. Being overwhelmed with parenting alone	○	○	○	○

The following questions come from a questionnaire that examines the thoughts and feelings of people who have lost someone important to them. In this study, the loss refers to the death of your co-parent. Please consider that loss as you respond to the following questions.

	Not at all	At least once	At least once a week	At least once a day	Several times a day
44. In the past month, how often have you felt yourself longing or yearning for the person you lost?	○	○	○	○	○
45. In the past month, how often have you had intense feelings of emotional pain, sorrow, or pangs of grief related to the lost relationship?	○	○	○	○	○

46. Have you experienced either of these symptoms at least daily and after 6 months have elapsed since the loss?	○ No<br>○ Yes

	Not at all	At least once	At least once a week	At least once a day	Several times a day
47. In the past month, how often have you tried to avoid reminders that the person you lost is gone?	○	○	○	○	○

48. In the past month, how often have you felt stunned, shocked, or dazed by your loss?	○	○	○	○	○

	Not at all	Slightly	Somewhat	Quite a bit	Overwhelmingly
49. Do you feel confused about your role in life or feel like you don't know who you are (i.e., feeling that a part of yourself has died)?	○	○	○	○	○
50. Have you had trouble accepting the loss?	○	○	○	○	○
51. Has it been hard for you to trust others since your loss?	○	○	○	○	○
52. Do you feel bitter over your loss?	○	○	○	○	○
53. Do you feel that moving on (e.g., making new friends, pursuing new interests) would be difficult for you now?	○	○	○	○	○
54. Do you feel emotionally numb since your loss?	○	○	○	○	○
55. Do you feel that life is unfulfilling, empty, or meaningless since your loss?	○	○	○	○	○

56. Have you experienced a significant reduction in social, occupational, or other important areas of functioning (e.g. domestic responsibilities)?	○ No<br>○ Yes

In the past 7 days…

	Never	Rarely	Sometimes	Often	Always
57. I felt worthless	○	○	○	○	○
58. I felt helpless	○	○	○	○	○
59. I felt depressed	○	○	○	○	○
60. I felt hopeless	○	○	○	○	○

Talking about the expected death of a parent with your child(ren) can be very challenging. We recognize that these conversations differ based on their age and ability to understand. We would like to know what your experience (if any) has been with this. ***Please think about your child(ren) who were under 18 when [Name] died.*** **[ONLY SHOW IF Q5 = Response 2-7]**

61. Before [*Name*] died, did either of you discuss with your child(ren) that [he/she] would not survive?	○ Yes, all of the children<br>○ Yes, some of the children<br>○ No, none of the children
62. Do you wish you had discussed this with your child(ren) before [*Name*] died?	○ Yes → SKIP TO NEXT SECTION<br>○ No → SKIP TO NEXT SECTION
63. About how long before [*Name*]'s death did one or both of you first discuss with your child(ren) that [*Name*] would not survive? [**ONLY SHOW IF Q62=NO]**	______Hours<br>______Days<br>______Weeks<br>______Months<br>______Years
64. Knowing what you know now, would you change anything about how you and/or [*Name*] communicated with your child(ren) about [*Name*]'s death?	○ Yes<br>○ No **[SKIP TO Q66]**
65. Please describe what you would like to have been different about this/these conversation(s)?	

Now we would like to ask about your communication about [Name] with your children since [he/she] died. Again, please think about your child(ren) who were under 18 when [Name] died. If you have more than one child, think of the child who has ***struggled the most*** *with the loss of [Name] when answering the questions below.*

66. How old is the child currently?	__________________ years
67. What is the child's gender?	○ Male<br>○ Female<br>○ Other, please describe
68. Please think about the past 30 days. On about how many of those days did you mention [*Name*] in conversation with your child, if any? Your best guess is fine.	______________days
69. In general, how comfortable do you feel talking about [*Name*] with your child?	○ Not at all<br>○ A little comfortable<br>○ Moderately comfortable<br>○ Very comfortable
70. In general, how comfortable do you believe your child feels talking with you about [*Name*]?	○ Not at all<br>○ A little comfortable<br>○ Moderately comfortable<br>○ Very comfortable
71. What, if anything, do you feel is the biggest challenge to talking about [*Name*] with your child?	

The statements below come from a questionnaire that describes children's behavior after a stressful experience. For the following questions, please continue to think about your child who has ***struggled the most*** *with the loss of [Name]. Please answer all items as well as you can even if some do not seem to apply to your child. In this study, the "event" refers to the death of your Name.*

Now *or* ***within the past month****…*

	Not true (as far as you know)	Somewhat or Sometimes True	Very True or Often True
72. Child gets very upset if reminded of the event	○	○	○
73. Child reports more physical complaints when reminded of the event. For example, headache, stomachache, nausea, difficulty breathing.	○	○	○
74. Child reports that he or she does not want to talk about the event.	○	○	○
75. Child startles easily. For example, he or she jumps when hears sudden or loud noises.	○	○	○

Thinking about your parenting in the time since [Name] died, how much would you disagree or agree with each of the following statements? Please select one response on each row.

	Strongly Disagree	Moderately Disagree	Mildly Disagree	Mildly Agree	Moderately Agree	Strongly Agree
76. I am doing an excellent job raising my child(ren) since [*Name*] died	○	○	○	○	○	○
77. I am unsure how to discipline my child(ren) since [*Name*] died	○	○	○	○	○	○
78. No matter how I raise my child(ren), it won't be good enough because they don't have another parent	○	○	○	○	○	○
79. I feel overwhelmed by the responsibilities of being a parent	○	○	○	○	○	○
80. [*Name*] would be proud of how I am raising our child(ren)	○	○	○	○	○	○

81. I am less strict with my child(ren) since [*Name*] died	○	○	○	○	○	○
82. Caring for my child(ren) takes up all my time and energy	○	○	○	○	○	○
83. My child(ren) listen to me as well as they did before [*Name*] died	○	○	○	○	○	○
84. I meet my own expectations for being a parent	○	○	○	○	○	○

Please use the scale below to indicate how you feel at the present time.

	Extremely Dissatisfied	Very Dissatisfied	Somewhat Dissatisfied	Mixed	Somewhat Satisfied	Very Satisfied	Extremely Satisfied
85. How satisfied are you with your child(ren)'s behavior?	○	○	○	○	○	○	○
86. How satisfied are you with yourself as a parent?	○	○	○	○	○	○	○
87. How satisfied are you with your relationship(s) with your child(ren)?	○	○	○	○	○	○	○

Next, we have some background questions for you.

88. What is your age?	_____ years
89. For how many years were you and [*Name*] together as a couple?	_____ years
90. At the time [*Name*] died, were you and [*Name*] still together as a couple?	○ Yes<br>○ No
91. Were you ever legally married to [*Name*]?	○ Yes<br>○ No
92. Are there still children under 18 in the home?	○ Yes<br>○ No
93. Would you say you are currently the sole caregiver for your child(ren), or is there another adult living in your household who shares responsibility for raising your child(ren)?	○ I am the sole caregiver<br>○ Another adult shares responsibility

94. How many children did you and [*Name*] have together who were under 18 when [*Name*] died? ________

95. For each child under 18 when [*Name*] died, please record the child's gender and his/her age at the time of [*Name*]'s death. **PROGRAMMER: DISPLAY NUMBER OF ROWS IN TABLE ACCORDING TO ANSWER IN Q94.**

	Gender<br>Male Female	Age when mother died<br>Age in Years
Child 1	◯ ◯	
Child 2	◯ ◯	
Child 3<br>etc.	◯ ◯	

96. How important or unimportant is religious faith in shaping how you live your daily life?
   - ○ Not important at all
   - ○ Somewhat important
   - ○ Very important

97. How important or unimportant is spirituality in shaping how you live your daily life?
   - ○ Not important at all
   - ○ Somewhat important
   - ○ Very important

98. These days, how much do you feel supported by friends, family, and/or other people in your life?
   - ○ Not supported at all
   - ○ Supported only a little
   - ○ Somewhat supported
   - ○ Strongly supported

99. How much help do you have from your friends/family for your parenting responsibilities?
   - ○ None
   - ○ A small amount
   - ○ A moderate amount
   - ○ A lot

100. How much help do you need from your friends/family for your parenting responsibilities?
   - ○ None
   - ○ A small amount
   - ○ A moderate amount
   - ○ A lot

101. How much contact have you had with other widowed parents like yourself?
   - ○ None
   - ○ A small amount
   - ○ A moderate amount
   - ○ A lot

102. How important is contact with other widowed parents for you?
   - ○ Not important at all
   - ○ Somewhat important
   - ○ Very important

103. What is your racial or ethnic background? *Check all that apply.*	○ Caucasian/White<br>○ African American/Black<br>○ Hispanic/Latino<br>○ Asian<br>○ Other racial/ethnic identity:
104. What is the highest level of education you have completed?	○ Some high school or less<br>○ High school graduate or GED<br>○ Some college<br>○ Associate's degree<br>○ College graduate<br>○ Postgraduate study or degree
105. What is your current employment status?	○ Employed full-time<br>○ Employed part-time<br>○ Full time student<br>○ Retired<br>○ Unemployed, looking for work<br>○ Other: please describe
106. What was your household income, before taxes, in the past 12 months?	○ Less than $25,000/year<br>○ $25,000 - $50,000/year<br>○ $50,000 - $100,000/year<br>○ Over $100,000/year
107. In what country do you live?	Country: [dropdown list of countries]

This is the end of the main part of our survey. If you can spare 3 or 4 more minutes, there is one last set of questions we would appreciate your input on. Would you be able to respond to one more set of questions?

Yes--> ADMINISTER MODULE 1 / MODULE 2

No--> Skip to Contact Info Request

MODULE 1 – DEPENDENCY ON THE BEREAVED

For each item below, please rate the extent to which each of the items is true with respect to [*Name*].

	1 (Not at all)	2	3 (Somewhat)	4	5 (Extremely)
1. How dependent were you on [*Name*] in general?	○	○	○	○	○
2. How dependent were you on [*Name*] emotionally?	○	○	○	○	○
3. How dependent were you on [*Name*] for household management?	○	○	○	○	○
4. How dependent were you on [*Name*] for getting around?	○	○	○	○	○
5. How dependent were you on [*Name*] socially?	○	○	○	○	○
6. How dependent were you on [*Name*] for maintaining your health?	○	○	○	○	○

MODULE 2 – CONTINUING BONDS SCALE

For each item below, please rate the extent to which each of the items is true with respect to [*Name*].

	1 (Not true at all)	2	3	4	5 (Very True)
1. I seek out things to remind me of [*Name*].	○	○	○	○	○
2. I keep items that belonged to or were closely associated with [*Name*] as a reminder of [him or her]	○	○	○	○	○
3. I like to reminisce with others about [*Name*].	○	○	○	○	○
4. I have inner conversations with [*Name*] where I turn to [him or her] for comfort or advice	○	○	○	○	○
5. Even though no longer physically present, [*Name]* continues to be a loving presence in my life	○	○	○	○	○
6. I am aware of having taken on many of [*Name*]'s habits, values, or interests	○	○	○	○	○
7. I am aware of the positive influence of [*Name*] on who I am today	○	○	○	○	○
8. I attempt to carry out [*Name*]'s wishes	○	○	○	○	○
9. I have many fond memories that bring joy to me	○	○	○	○	○
10. When making decisions, I imagine [*Name*]'s viewpoint and use this as a guide in deciding what to do	○	○	○	○	○
11. I experience [*Name*] as continuing to live on through me	○	○	○	○	○
