# Supplementary figures and images for: Parenting through grief: A cross-sectional study of recently bereaved adults with minor children
Source: Palliat Med. 2021 Aug 22;35(10):1923–32. doi: 10.1177/02692163211040982 (PMC8637383; doi:10.1177/02692163211040982)

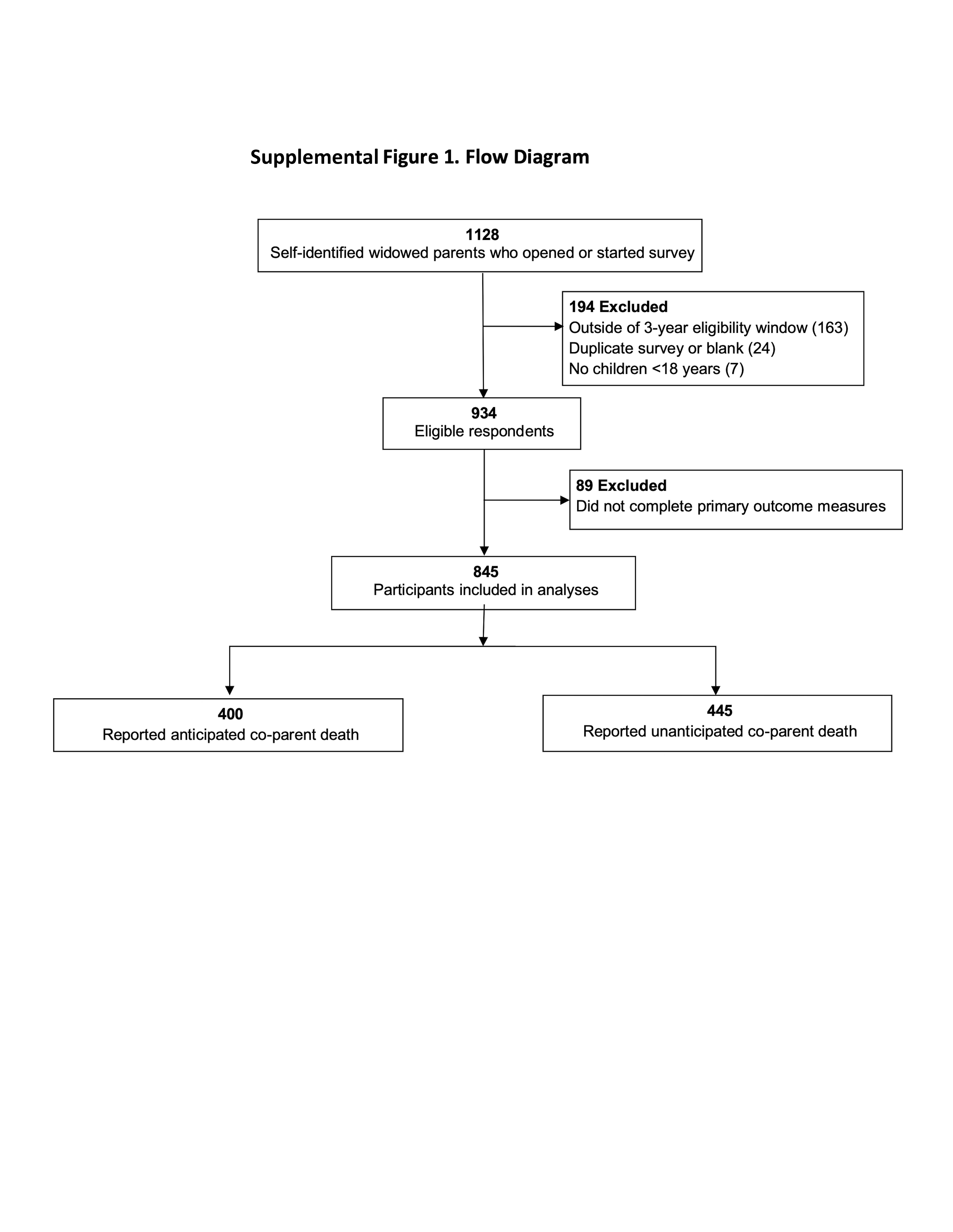

Supplement: sj-tif-2-pmj-10.1177_02692163211040982 – Supplemental material for Parenting through grief: A cross-sectional study of recently bereaved adults with minor children [file sj-tif-2-pmj-10.1177_02692163211040982.tif]
